# Exploratory evaluation of combined handgrip strength and Age-Adjusted Charlson Comorbidity Index for prognosis in patients with primary liver cancer undergoing hepatectomy: a single-centre pilot study

**DOI:** 10.3389/fmed.2026.1875463

**Published:** 2026-09-08

**Authors:** Dongqing Wang, Yan Cheng, Mao Chen, Junying Li, Xiaoliang Kang

**Affiliations:** 1 Department of Emergency Medicine, The First Affiliated Hospital of Chengdu Medical College, Chengdu, China; 2 Department of Hematology, The First Affiliated Hospital of Chengdu Medical College, Chengdu, China; 3 Department of Hepatobiliary and Vascular Surgery, The First Affiliated Hospital of Chengdu Medical College, Chengdu, China

**Keywords:** Age-Adjusted Charlson Comorbidity Index, handgrip strength, overall survival, primary liver cancer, recurrence-free survival

## Abstract

**Objective:**

To investigate whether preoperative handgrip strength and the Age-Adjusted Charlson Comorbidity Index (ACCI), alone and in combination, predict overall survival and recurrence-free survival in patients with primary liver cancer undergoing curative-intent hepatectomy.

**Materials and methods:**

This prospective single-centre pilot study consecutively enrolled patients with pathologically confirmed primary liver cancer who underwent curative-intent hepatectomy at The First Affiliated Hospital of Chengdu Medical College between January 2020 and December 2022. Handgrip strength was measured preoperatively using a calibrated dynamometer. ACCI was calculated from documented comorbidities with age adjustment. Overall survival and recurrence-free survival were estimated by Kaplan–Meier analysis and compared by log-rank testing. Cox proportional hazards models were used to identify independent prognostic factors.

**Results:**

A total of 1,134 patients were screened, and 783 patients were ultimately included in this study. Low handgrip strength was more frequent in females, patients aged >65 years, higher tumor stage, and those with reduced total protein, and reduced albumin. High ACCI was more frequently observed in patients with hypertension, diabetes, elevated AST, elevated ALT, reduced total protein, and reduced albumin. In multivariable analyses, tumor stage, handgrip strength, and ACCI were independent predictors of overall survival and recurrence-free survival. Patients with concomitantly low handgrip strength and high ACCI had significantly poorer overall survival and recurrence-free survival compared with those with high handgrip strength and low ACCI. The combined handgrip strength and ACCI model provided preliminary evidence of incremental prognostic discrimination compared with either marker alone.

**Conclusion:**

Preoperative handgrip strength and ACCI independently predict overall survival and recurrence-free survival after hepatectomy for primary liver cancer, and their combination improves prognostic stratification, which may offer a tool for perioperative risk assessment and individualized surveillance planning.

## Introduction

Primary liver cancer, predominantly hepatocellular carcinoma (HCC) and intrahepatic cholangiocarcinoma (ICC), remains a leading cause of cancer-related mortality worldwide (1–3). Liver cancer accounted for a substantial proportion of global cancer deaths. For appropriately selected patients, hepatectomy is a cornerstone curative-intent treatment and can confer durable survival benefits; however, postoperative morbidity and heterogeneous long-term outcomes remain common (4). Despite advances in surgical techniques and perioperative care, complications and recurrence after surgery continue to adversely affect prognosis (5). Therefore, simple and robust tools are needed to evaluate perioperative risk and identify patients most likely to benefit from hepatectomy.

Beyond tumor biology, baseline comorbidity burden strongly influences surgical tolerance, competing-risk mortality, and cancer-specific outcomes. The Age-Adjusted Charlson Comorbidity Index (ACCI) integrates chronic disease burden with age and has demonstrated prognostic value after liver resection (6). Among patients with ICC undergoing curative resection, a higher ACCI was independently associated with worse overall survival (6). Similarly, published research indicated that ACCI predicted both short- and long-term outcomes after hepatic resection for HCC, supporting its role in surgical decision-making and perioperative planning (7).

Sarcopenia and impaired physical function represent another important and potentially modifiable dimension of surgical risk (8, 9). Handgrip strength is an inexpensive, noninvasive surrogate of muscle strength and has been associated with adverse outcomes in liver cancer (10, 11). In a multicenter cohort of patients with liver cancer, low handgrip strength was associated with increased mortality, and the combination of low handgrip strength and a high modified Glasgow Prognostic Score further amplified risk (12). Recent studies also suggest that sarcopenia definitions incorporating handgrip strength can predict postoperative complications after hepatectomy for HCC (13).

Despite these advances, few studies have examined whether integrating functional reserve (handgrip strength) with systemic comorbidity burden (ACCI) improves prognostic stratification specifically in patients with primary liver cancer undergoing hepatectomy. Building on evidence that comprehensive perioperative assessment can improve recovery after hepatectomy, we propose a pragmatic prognostic framework that integrates handgrip strength and the ACCI to predict survival and recurrence after hepatectomy. This model may facilitate preoperative risk assessment, guide perioperative management, and inform personalized postoperative surveillance strategies.

## Patients and methods

### Study patients

This prospective single-centre pilot study consecutively enrolled patients with primary liver cancer undergoing hepatectomy at The First Affiliated Hospital of Chengdu Medical College between January 2020 and December 2022. Patients were screened at the time of surgical evaluation and were eligible if they, (1) were adults (≥18 years); (2) underwent curative-intent hepatectomy for primary liver cancer; (3) had postoperative pathological confirmation of primary liver cancer including HCC and/or ICC; and (4) preserved preoperative liver function (Child–Pugh Class A). Exclusion criteria included, (1) evidence of extrahepatic metastasis at baseline evaluation; (2) palliative/non-curative resection; (3) concomitant local ablation or major surgery of other organs during the same operation; (4) inability to complete preoperative handgrip strength assessment (e.g., severe upper-limb dysfunction or neurologic disease precluding reliable measurement); and (5) missing key baseline data needed to calculate handgrip strength or ACCI.

All procedures were conducted in accordance with the Declaration of Helsinki. Written informed consent was obtained from all participants before enrollment, and the study was approved by the ethics committee of The First Affiliated Hospital of Chengdu Medical College (No. 10342MD273).

### Clinical variables

Baseline and perioperative data were prospectively collected using standardized case-report forms and cross-checked against the electronic medical records. Consistent with established reporting in hepatectomy studies, collected variables covered, (1) demographics and general condition (age, sex, body mass index, smoking/drinking history, performance status, and anesthetic risk as applicable); (2) comorbidities and medication history; (3) laboratory tests (complete blood count, liver function tests, renal function, coagulation profile, inflammatory and nutritional markers), and tumor markers; (4) tumor-related characteristics from preoperative imaging and postoperative pathology (tumor size, number, location, differentiation, microvascular invasion, resection margin status); and (5) operative and perioperative factors (surgical approach, extent of resection, operative time, blood loss/transfusion, and postoperative complications).

### Handgrip strength assessment

Handgrip strength was measured preoperatively (typically within 7 days before surgery) by trained staff using a calibrated handheld dynamometer. Measurements were performed with the patient seated, shoulder neutrally positioned, elbow flexed at 90°, and wrist in a neutral position. Patients performed three maximal-effort trials on the dominant hand with standardized verbal encouragement and adequate rest between attempts; the highest value (kg) was used for analysis (14).

The log-rank method was applied to identify the cutoff values for handgrip strength separately for male and female participants. The optimal thresholds were determined to be 26.8 for men and 17.6 for women. Based on these values, patients were categorized into either low or high handgrip strength groups.

### Age-Adjusted Charlson Comorbidity Index calculation

Comorbidities were recorded from patient interviews, pre-anesthesia assessment, and prior medical documentation, then mapped to Charlson Comorbidity Index (CCI) conditions. The CCI score was calculated by identifying the comorbid conditions present in each patient and assigning a weighted score according to the predefined categories in the CCI table. Each medical condition was given a specific weight reflecting its relative impact on mortality risk. Conditions including myocardial infarction, congestive heart failure, peripheral vascular disease, dementia, cerebrovascular disease, chronic pulmonary disease, ulcer disease, diabetes, hypertension, and mild liver disease were assigned 1 point each. Moderate or severe renal disease, hemiplegia, malignant lymphoma, and any tumor were assigned 2 points each. Moderate or severe liver disease was assigned 3 points, whereas metastatic solid tumor and acquired immune deficiency syndrome were assigned 6 points. For each patient, the points corresponding to all documented comorbid conditions were summed to obtain the total CCI score. The ACCI was calculated by adding age points to the CCI score, for patients over 40 years, extra points was added to the final CCI scores (1 point for 50–59 years, 2 points for 60–69 years, 3 points for 70–79 years and 4 points for ≥80 years) (15). In the current study, the cutoff value of 5 points was determined based on the median ACCI values of the study participants. Based on the cutoff value, patients were categorized into either low or high ACCI groups.

### Follow-up

All patients were followed from the date of hepatectomy until death, and the last follow-up date was December 31, 2025. Follow-up information was obtained through scheduled outpatient visits and, when necessary, structured telephone interviews, consistent with established approaches for outcome ascertainment in liver cancer cohorts.

Patients were generally assessed every 3 months during the first 2 years after surgery and every 6 months thereafter. At each follow-up, clinical evaluation and routine laboratory testing were performed, including liver function tests and tumor markers. Imaging surveillance was conducted using abdominal ultrasonography and/or contrast-enhanced CT or MRI according to clinical practice and guideline-based surveillance after curative-intent resection.

Overall survival was defined as the time from hepatectomy to death from any cause or the last documented follow-up, and patients alive at the last follow-up were censored. Recurrence-free survival was defined as the time from hepatectomy to the first documented tumor recurrence or death from any cause, whichever occurred first, and patients without recurrence were censored at the date of last imaging confirming recurrence-free status. Tumor recurrence was diagnosed based on radiologic evidence on dynamic contrast-enhanced CT/MRI (and/or histopathology when clinically indicated).

### Statistical analysis

Continuous variables were summarized as mean ± standard deviation (SD) for normally distributed data or median (IQR, interquartile range) for non-normally distributed data, categorical variables were presented as numbers (percentages). Between-group comparisons were performed using Student's *t* test or Wilcoxon rank-sum test for continuous variables and the *χ*^2^ test (or Fisher's exact test, as appropriate) for categorical variables.

Survival curves for overall survival and recurrence-free survival were estimated using the Kaplan–Meier method and compared using the log-rank test. Cox proportional hazards regression was used to evaluate associations of handgrip strength, ACCI, and their combined model with overall survival and recurrence-free survival, reporting hazard ratios (HRs) with 95% confidence intervals (CIs). Multivariable models were constructed with stepwise adjustment for clinically relevant confounders, following a tiered modeling strategy used in comparable liver cancer prognostic studies.

Receiver operating characteristic (ROC) curves were generated to evaluate the prognostic discrimination of handgrip strength, ACCI, and the combined handgrip strength–ACCI model. AUC values with 95% confidence intervals, sensitivity, specificity, and statistical comparisons were reported. Time-dependent AUCs with DeLong tests were used to estimate the discriminative ability of handgrip strength, ACCI, and the combined handgrip strength–ACCI model at 1, 2, 3, 4, and 5 years. All statistical analyses were performed using SPSS (SPSS, Inc., Chicago, United States) and two-sided *p* values <0.05 were considered statistically significant.

## Results

### Patient characteristics

Initially, this research screened 1,134 primary liver cancer patients. After excluding 351 patients who didn't meet the inclusion and exclusion criteria, a total of 783 patients with primary liver cancer undergoing hepatectomy were included. As shown in Table 1, hepatocellular carcinoma was the predominant histology (84.93%), whereas intrahepatic cholangiocarcinoma accounted for 118 cases (15.07%). The cohort was mainly male (74.71%), and more than half of patients were aged 65 years or younger (55.81%). Regarding tumor stage, most patients had stage II disease (57.09%), followed by stage I (31.03%) and stage IIIa (11.88%).

**Table 1:** Patient characteristics of the study population.

Patient characteristics	*n*	Percentage (%)
Total	783	100
Histology type		
HCC	665	84.93
ICC	118	15.07
Gender		
Male	585	74.71
Female	198	25.29
Age (years)		
≤65	437	55.81
>65	346	44.19
Tumor stage		
I	243	31.03
II	447	57.09
IIIa	93	11.88
Hypertension		
No	659	84.16
Yes	124	15.84
Diabetes		
No	735	93.87
Yes	48	6.13
AST		
Normal	652	83.27
Elevated	131	16.73
ALT		
Normal	613	78.29
Elevated	170	21.71
Total protein		
Normal	665	84.93
Reduced	118	15.07
Albumin		
Normal	671	85.70
Reduced	112	14.30
AFP		
Normal	81	10.34
Elevated	702	89.66
CA 19-9		
Normal	654	83.52
Elevated	129	16.48
CEA		
Normal	707	90.29
Elevated	76	9.71
Handgrip strength		
Low	432	55.17
High	351	44.83
ACCI		
Low	365	46.62
High	418	53.38

ACCI, age-adjusted Charlson Comorbidities Index; AFP, *α*-fetoprotein; ALT, alanine aminotransferase; AST, aspartate transaminase; CA19-9, carbohydrate 19-9; CEA, carcinoembryonic antigen; HCC, hepatocellular carcinoma; ICC, intrahepatic cholangiocarcinoma.

In terms of comorbidities, 124 patients (15.84%) had hypertension, and 48 patients (6.13%) had diabetes. Liver enzyme abnormalities were present in a subset of patients, with abnormal AST in 131 patients (16.73%) and abnormal ALT in 170 patients (21.71%). Most patients had normal total protein (84.93%) and albumin levels (85.70%). For tumor markers, AFP was elevated in 702 patients (89.66%), whereas CA19-9 and CEA were elevated in 129 (16.48%) and 76 (9.71%) patients, respectively. With respect to functional reserve and comorbidity burden, low handgrip strength was observed in 432 patients (55.17%), and high ACCI score was present in 418 patients (53.38%).

### The association between handgrip strength and patient clinicopathologic characteristics

The associations between handgrip strength and patient clinicopathologic characteristics are summarized in Table 2. Handgrip strength was significantly associated with gender (*p* < 0.0001) and age (*p* < 0.0001), females and patients aged >65 years were more likely to have low handgrip strength. Handgrip strength also differed across tumor stages (*p* < 0.0001). Specifically, low handgrip strength was more commonly observed in stage II and IIIa disease. In addition, patients with reduced total protein (*p* = 0.010) and albumin (*p* < 0.0001) more often have low handgrip strength.

**Table 2:** The association between handgrip strength and patient characteristics.

Patient characteristics	Handgrip Strength		*p*-value
	Low (432)	High (351)	
Histology Type			0.104
HCC	375	290	
ICC	57	61	
Gender			**<0** **.** **0001**
Male	268	317	
Female	164	34	
Age (years)			**<0** **.** **0001**
≤65	203	234	
>65	229	117	
Tumor stage			**<0** **.** **0001**
I	53	190	
II	306	141	
IIIa	73	20	
Hypertension			0.272
No	358	301	
Yes	74	50	
Diabetes			0.098
No	400	335	
Yes	32	16	
AST			0.195
Normal	353	299	
Elevated	79	52	
ALT			0.313
Normal	344	269	
Elevated	88	82	
Total protein			**0** **.** **010**
Normal	354	311	
Reduced	78	40	
Albumin			**<0** **.** **0001**
Normal	347	324	
Reduced	85	27	
AFP			0.136
Normal	51	30	
Elevated	381	321	
CA 19-9			0.419
Normal	365	289	
Elevated	67	62	
CEA			0.231
Normal	395	312	
Elevated	37	39	

AFP, *α*-fetoprotein; ALT, alanine aminotransferase; AST, aspartate transaminase; CA19-9, carbohydrate 19-9; CEA, carcinoembryonic antigen; HCC, hepatocellular carcinoma; ICC, intrahepatic cholangiocarcinoma.

The Chi-square tests were used for statistical analyses, and *p* values <0.05 were considered statistically significant and highlighted in bold text.

No statistically significant associations with handgrip strength were found for tumor histology types, hypertension or diabetes, liver enzymes (AST, ALT), or tumor markers including AFP, CA19-9, and CEA (all *p* > 0.05).

### The association between ACCI and patient clinicopathologic characteristics

The associations between ACCI and patient clinicopathologic characteristics are summarized in Table 3. Age and tumor stage are significantly associated with ACCI, patients with elder age (>65 years) and higher tumor stage had higher ACCI scores.

**Table 3:** The association between Age-Adjusted Charlson Comorbidity Index and patient characteristics.

Patient characteristics	ACCI		*p*-value
	Low (365)	High (418)	
Histology type			0.231
HCC	288	377	
ICC	77	41	
Gender			0.204
Male	265	320	
Female	100	98	
Age (years)			**<0** **.** **0001**
≤65	235	202	
>65	130	216	
Tumor stage			**<0** **.** **0001**
I	160	83	
II	172	275	
IIIa	33	60	
Hypertension			**<0** **.** **0001**
No	345	314	
Yes	20	104	
Diabetes			**0** **.** **005**
No	352	383	
Yes	13	35	
AST			**0** **.** **0015**
Normal	353	299	
Elevated	51	80	
ALT			**0** **.** **0002**
Normal	307	306	
Elevated	58	112	
Total protein			**0** **.** **009**
Normal	323	342	
Reduced	42	76	
Albumin			**<0** **.** **0001**
Normal	349	322	
Reduced	16	96	
AFP			0.446
Normal	41	40	
Elevated	324	378	
CA 19-9			0.057
Normal	295	359	
Elevated	70	59	
CEA			0.269
Normal	325	382	
Elevated	40	36	

ACCI, age-adjusted Charlson Comorbidities Index; AFP, *α*-fetoprotein; ALT, alanine aminotransferase; AST, aspartate transaminase; CA19-9, carbohydrate 19-9; CEA, carcinoembryonic antigen; HCC, hepatocellular carcinoma; ICC, intrahepatic cholangiocarcinoma.

The Chi-square tests were used for statistical analyses, and *p* values <0.05 were considered statistically significant and highlighted in bold text.

Comorbidity profiles were consistent with the ACCI stratification. Hypertension (*p* < 0.0001) and diabetes (*p* = 0.005) were more prevalent in the high-ACCI group. In addition, high ACCI was significantly associated with abnormal liver biochemistry and nutritional indicators, including elevated AST (*p* = 0.0015) and elevated ALT (*p* = 0.0002), reduced total protein (*p* = 0.009), and reduced albumin (*p* < 0.0001). By contrast, ACCI was not significantly associated with tumor histology type, gender, AFP, CA 19-9, or CEA (all *p* > 0.05).

### Identification of risk factors for overall survival and recurrence-free survival in patients with primary liver cancer

We performed univariate and multivariate Cox proportional hazards regression analyses to determine prognostic factors in patients with primary liver cancer. The univariate analysis showed that factors like age, tumor stage, AST, ALT, total protein, albumin, handgrip strength, and ACCI score were risk factors for overall survival rates (Table 4). When adjusting for potential confounding factors, including histology type, gender, age, tumor stage, hypertension, diabetes, AST, ALT, total protein, albumin, AFP, CA 19-9, CEA, handgrip strength, and ACCI score, the multivariate analysis identified the tumor stage (HR = 2.06, 95% Cl, 1.03–3.54, *p* < 0.0001), handgrip strength (HR = 2.67, 95% Cl, 1.36–4.92, *p* = 0.001), and ACCI score (HR = 3.23, 95% Cl, 2.21–4.51, *p* < 0.0001) as independent risk factors for overall survival rates (Table 4).

**Table 4:** Identification of risk factors for overall survival in patients with primary liver cancer.

Patient characteristics	Univariate analysis	*p*-value	Multivariate analysis	*p*-value
	HR (95% CI)		HR (95% CI)	
Histology Type				
HCC	1.00 (Reference)		1.00 (Reference)	
ICC	0.93 (0.63–0.95)	0.131	0.85 (0.72–0.91)	0.241
Gender				
Male	1.00 (Reference)		1.00 (Reference)	
Female	1.02 (1.01–1.34)	0.176	1.12 (1.07–1.41)	0.235
Age (years)				
≤65	1.00 (Reference)		1.00 (Reference)	
>65	2.82 (1.76–4.42)	0.004	1.24 (1.02–1.83)	0.244
Tumor stage				
I	1.00 (Reference)		1.00 (Reference)	
II	2.25 (1.13–3.89)	**<0** **.** **0001**	2.06 (1.03–3.54)	**<0** **.** **0001**
IIIa	4.32 (2.31–6.43)	**<0** **.** **0001**	4.87 (2.52–6.83)	**<0** **.** **0001**
Hypertension				
No	1.00 (Reference)		1.00 (Reference)	
Yes	1.01 (1.00–1.09)	0.165	1.02 (1.00–1.37)	0.219
Diabetes				
No	1.00 (Reference)		1.00 (Reference)	
Yes	1.22 (1.04–1.47)	0.246	1.08 (1.03–1.32)	0.225
AST				
Normal	1.00 (Reference)		1.00 (Reference)	
Elevated	2.37 (1.25–4.18)	**0** **.** **001**	1.04 (1.01–1.32)	0.071
ALT				
Normal	1.00 (Reference)		1.00 (Reference)	
Elevated	2.53 (1.32–4.35)	**0** **.** **002**	1.13 (1.02–1.61)	0.103
Total protein				
Normal	1.00 (Reference)		1.00 (Reference)	
Reduced	2.72 (1.04–4.41)	**0** **.** **003**	1.08 (1.01–0.19)	0.172
Albumin				
Normal	1.00 (Reference)		1.00 (Reference)	
Reduced	3.45 (1.31–5.65)	**0** **.** **001**	1.25 (1.02–2.98)	0.218
AFP				
Normal	1.00 (Reference)		1.00 (Reference)	
Elevated	1.04 (1.03–1.32)	0.321	1.02 (1.01–1.09)	0.276
CA 19-9				
Normal	1.00 (Reference)		1.00 (Reference)	
Elevated	1.03 (1.01–1.07)	0.228	1.05 (1.04–1.13)	0.394
CEA				
Normal	1.00 (Reference)		1.00 (Reference)	
Elevated	1.11 (1.02–1.42)	0.253	1.02 (1.01–1.08)	0.425
Handgrip strength				
High	1.00 (Reference)		1.00 (Reference)	
Low	3.54 (2.22–4.87)	**0** **.** **002**	2.67 (1.36–4.92)	**0** **.** **001**
ACCI				
Low	1.00 (Reference)		1.00 (Reference)	
High	3.54 (2.35–4.82)	**<0** **.** **0001**	3.23 (2.21–4.51)	**<0** **.** **0001**

ACCI, age-adjusted Charlson Comorbidities Index; AFP, *α*-fetoprotein; ALT, alanine aminotransferase; AST, aspartate transaminase; CA19-9, carbohydrate 19-9; CEA, carcinoembryonic antigen; CI, confidential interval; HCC, hepatocellular carcinoma; HR, hazard ratio; ICC, intrahepatic cholangiocarcinoma.

Univariate and Multivariable models were constructed with stepwise adjustment for clinically relevant confounders, following a tiered modeling strategy used in comparable liver cancer prognostic studies to identify risk factors. A *p* value <0.05 was considered statistically significant and highlighted in bold text.

We further evaluated recurrence-free survival rates, and found that factors like tumor stage, AST, ALT, handgrip strength, and ACCI were risk factors for recurrence-free survival rates (Table 5). Upon adjusting for confounding factors, including histology type, gender, age, tumor stage, hypertension, diabetes, AST, ALT, total protein, albumin, AFP, CA 19-9, CEA, handgrip strength, and ACCI score, only the tumor stage (HR = 2.38, 95% Cl, 1.24–3.98, *p* < 0.0001), handgrip strength (HR = 2.65, 95% Cl, 1.15–4.85, *p* = 0.002), and ACCI score (HR = 3.01, 95% Cl, 2.07–4.95, *p* < 0.0001) emerged as independent risk factors for recurrence-free survival rates (Table 5).

**Table 5:** Identification of risk factors for recurrence-free survival in patients with primary liver cancer.

Patient characteristics	Univariate analysis	*p*-value	Multivariate analysis	*p*-value
	HR (95% CI)		HR (95% CI)	
Histology type				
HCC	1.00 (Reference)		1.00 (Reference)	
ICC	1.15 (1.03–1.38)	0.226	1.05 (1.01–1.28)	0.161
Gender				
Male	1.00 (Reference)		1.00 (Reference)	
Female	1.03 (1.01–1.27)	0.104	1.02 (1.01–1.02)	0.331
Age (years)				
≤65	1.00 (Reference)		1.00 (Reference)	
>65	1.02 (1.01–1.34)	0.424	1.04 (1.00–1.32)	0.175
Tumor stage				
I	1.00 (Reference)		1.00 (Reference)	
II	3.17 (2.14–4.76)	**<0** **.** **0001**	2.38 (1.24–3.98)	**<0** **.** **0001**
IIIa	4.56 (2.45–6.83)	**<0** **.** **0001**	4.83 (2.24–6.27)	**<0** **.** **0001**
Hypertension				
No	1.00 (Reference)		1.00 (Reference)	
Yes	1.03 (1.01–1.05)	0.263	1.07 (1.03–1.24)	0.112
Diabetes				
No	1.00 (Reference)		1.00 (Reference)	
Yes	1.14 (1.04–1.31)	0.445	1.04 (1.01–1.28)	0.371
AST				
Normal	1.00 (Reference)		1.00 (Reference)	
Elevated	2.48 (1.31–4.25)	**0** **.** **002**	1.06 (1.03–1.36)	0.152
ALT				
Normal	1.00 (Reference)		1.00 (Reference)	
Elevated	2.33 (1.16–3.21)	**0** **.** **003**	1.04 (1.02–1.14)	0.215
Total protein				
Normal	1.00 (Reference)		1.00 (Reference)	
Reduced	1.04 (1.01–1.13)	0.311	1.02 (1.00–1.08)	0.129
Albumin				
Normal	1.00 (Reference)		1.00 (Reference)	
Reduced	1.12 (1.02–1.34)	0.347	1.15 (1.08–1.28)	0.422
AFP				
Normal	1.00 (Reference)		1.00 (Reference)	
Elevated	1.02 (1.01–1.27)	0.489	1.04 (1.01–1.15)	0.316
CA 19-9				
Normal	1.00 (Reference)		1.00 (Reference)	
Elevated	1.03 (1.00–1.32)	0.268	1.02 (1.00–1.16)	0.187
CEA				
Normal	1.00 (Reference)		1.00 (Reference)	
Elevated	1.14 (1.02–1.31)	0.151	1.03 (1.02–1.13)	0.332
Handgrip Strength				
High	1.00 (Reference)		1.00 (Reference)	
Low	3.53 (2.43–4.91)	**0** **.** **001**	2.65 (1.15–4.85)	**0** **.** **002**
ACCI				
Low	1.00 (Reference)		1.00 (Reference)	
High	3.68 (2.17–4.97)	**<0** **.** **0001**	3.01 (2.07–4.95)	**<0** **.** **0001**

ACCI, age-adjusted Charlson Comorbidities Index; AFP, *α*-fetoprotein; ALT, alanine aminotransferase; AST, aspartate transaminase; CA19-9, carbohydrate 19-9; CEA, carcinoembryonic antigen; CI, confidential interval; HCC, hepatocellular carcinoma; HR, hazard ratio; ICC, intrahepatic cholangiocarcinoma.

Univariate and Multivariable models were constructed with stepwise adjustment for clinically relevant confounders, following a tiered modeling strategy used in comparable liver cancer prognostic studies to identify risk factors. A *p* value <0.05 was considered statistically significant and highlighted in bold text.

### Survival differences among patients with primary liver cancer stratified by handgrip strength and ACCI

**Figure 1:**
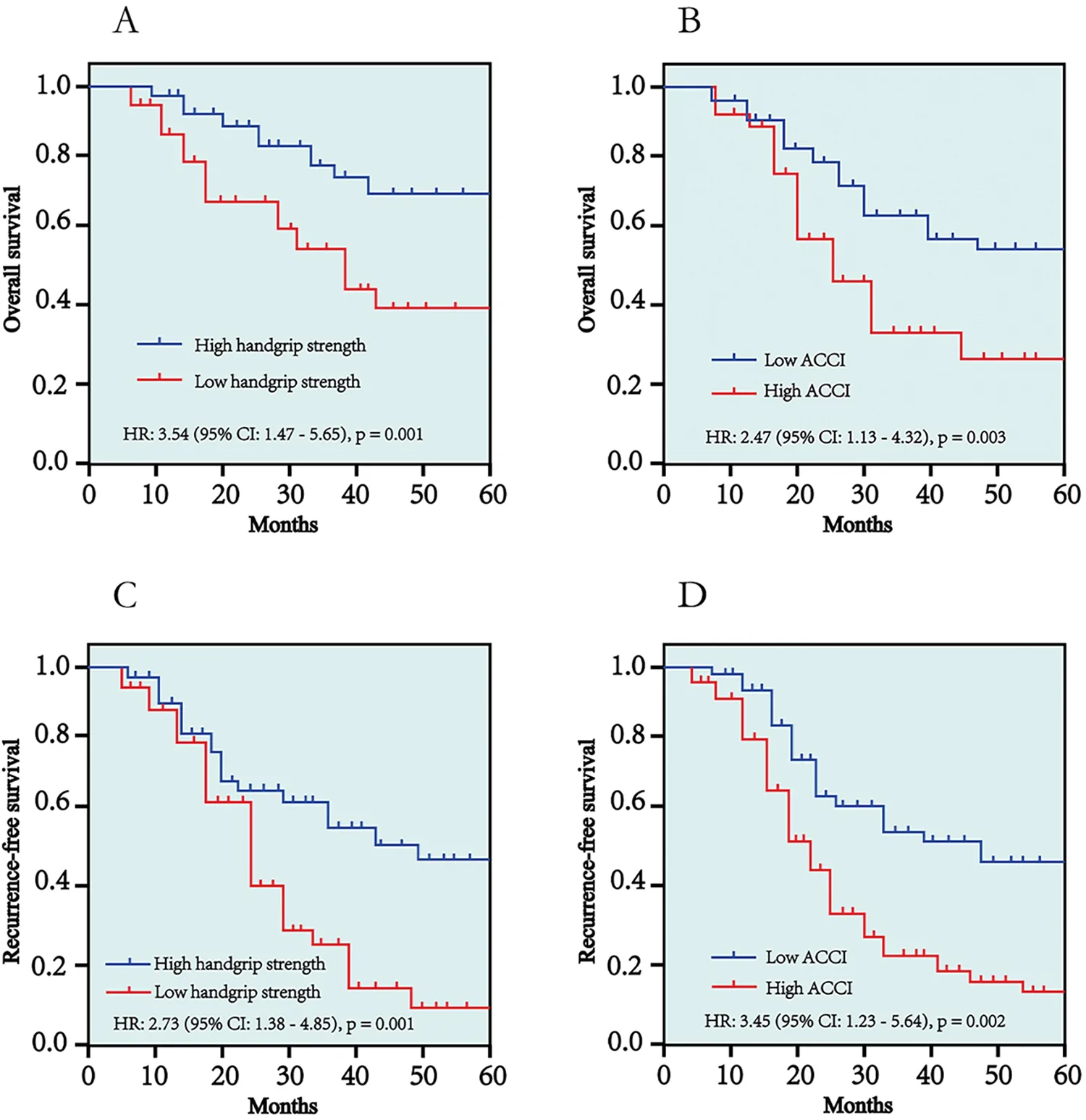
The Kaplan–Meier curves were used to compare the differences of overall survival **(A,B)** and recurrence-free survival (**C,D**) in patients with different handgrip strength and ACC scores. A *p* < 0.05 was considered statistically significant. ACCI, Age-Adjusted Charlson Comorbidity Index; CI, confidence interval; HR, hazard ratio.

The above findings indicated that handgrip strength and ACCI were independent risk factors for both overall survival and recurrence-free survival in patients with primary liver cancer. Kaplan–Meier analyses were subsequently performed to compare survival outcomes among groups stratified by handgrip strength and ACCI. Patients with low handgrip strength and a high ACCI score had significantly poorer overall survival than those with high handgrip strength (HR, 3.54; 95% CI, 1.47–5.65; *p* = 0.001) and a low ACCI score (HR, 2.47; 95% CI, 1.13–4.32; *p* = 0.003) (Figures 1A,B). Similarly, recurrence-free survival was lower in patients with low handgrip strength and a high ACCI score compared with those with high handgrip strength (HR, 2.73; 95% CI, 1.38–4.85; *p* = 0.001) and a low ACCI score (HR, 3.45; 95% CI, 1.23–5.64; *p* = 0.002) (Figures 1C,D).

### The performance of handgrip strength and ACCI in the prediction of overall survival and recurrence-free survival of patients with primary liver cancer

**Figure 2:**
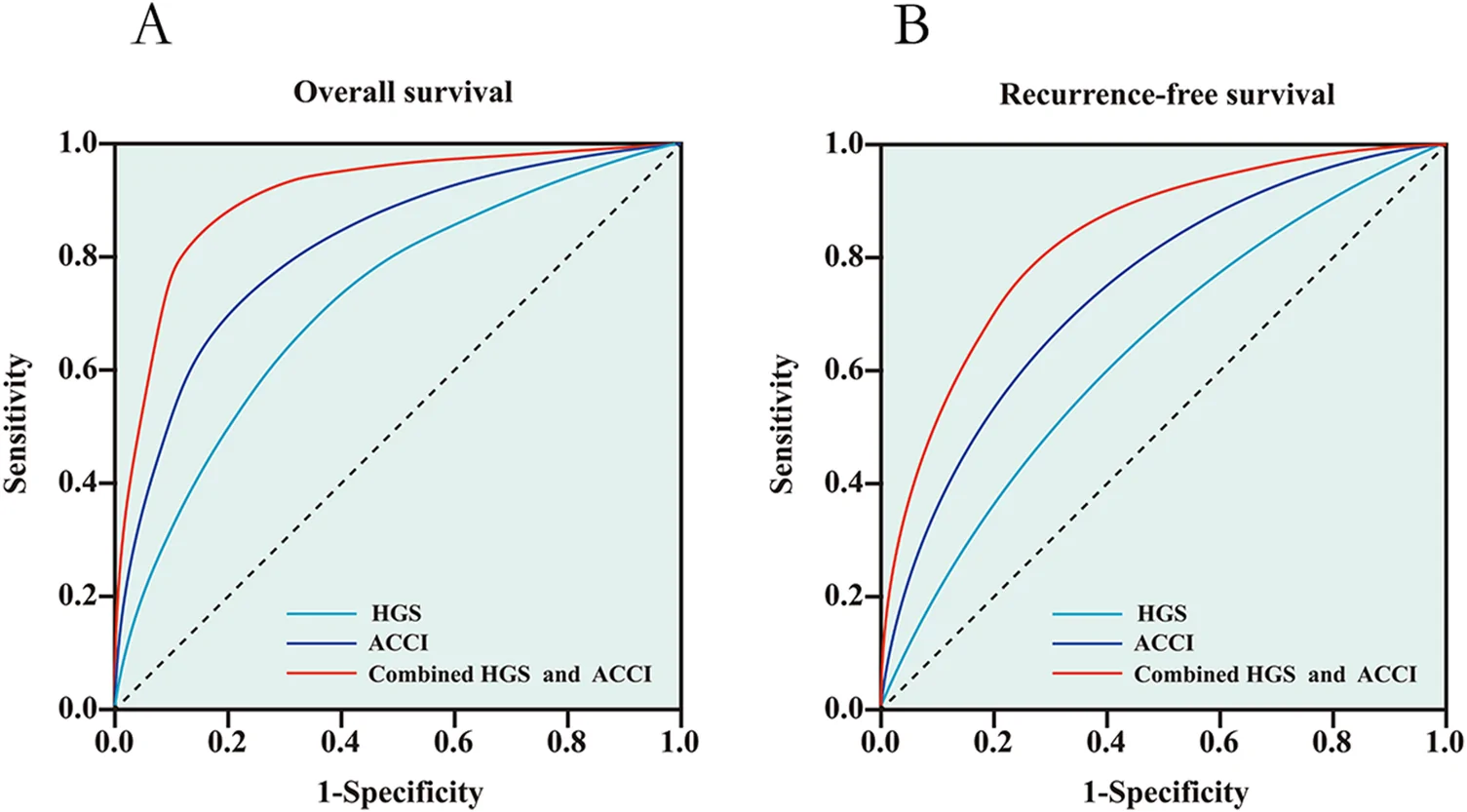
Receiver operating characteristic curve analyses were performed to estimate the performance of handgrip strength and ACCI in the prediction of overall survival **(A)** and recurrence-free survival **(B)** of patients with primary liver cancer. **(A)** The predictive performance of overall survival was 0.73 (95% CI, 0.62–0.82; *p* = 0.001), 0.81 (95% CI, 0.67–0.95; *p* = 0.002), and 0.86 (95% CI, 0.73–0.92; *p* < 0.0001) for handgrip strength, ACCI, and combined handgrip strength and ACCI, respectively. **(B)** The predictive performance of recurrence-free survival was 0.65 (95% CI, 0.52–0.81; *p* = 0.002), 0.75 (95% CI, 0.62–0.88; *p* = 0.004), and 0.81 (95% CI, 0.76–0.91; *p* < 0.0001) for handgrip strength, ACCI, and combined handgrip strength and ACCI, respectively. A *p* < 0.05 was considered statistically significant. ACCI, Age-Adjusted Charlson Comorbidity Index; AUC, area under the curve; CI, confidence interval; HGS, handgrip strength.

The ROC curve analysis was performed to calculate the AUC, sensitivity, and specificity, thereby assessing the ability of handgrip strength and the ACCI to predict overall survival and recurrence-free survival in patients with primary liver cancer. The combined model incorporating handgrip strength and ACCI achieved the highest AUC for both outcomes, with an AUC of 0.86 (95% CI, 0.73–0.92; *p* < 0.0001) for overall survival and 0.81 (95% CI, 0.76–0.91; *p* < 0.0001) for recurrence-free survival (Figure 2 and Table 6). This combination demonstrated better discriminative performance for both overall survival and recurrence-free survival than either handgrip strength or ACCI alone.

**Table 6:** The performance of handgrip strength and ACCI in the prediction of overall survival and recurrence-free survival of patients with primary liver cancer.

Biomarkers	Predictive performance of overall survival				Predictive performance of recurrence-free survival			
	AUC (95% CI)	Sensitivity	Specificity	*p*-value	AUC (95% CI)	Sensitivity	Specificity	*p*-value
HGS	0.73 (0.62–0.82)	65.61%	68.24%	**0** **.** **001**	0.65 (0.52–0.81)	61.78%	64.89%	**0** **.** **002**
ACCI	0.81 (0.67–0.95)	72.34%	74.36%	**0** **.** **002**	0.75 (0.62–0.88)	74.27%	73.45%	**0** **.** **004**
HGS + ACCI	0.86 (0.73–0.92)	81.23%	83.27%	**<0** **.** **0001**	0.81 (0.76–0.91)	80.54%	82.62%	**<0** **.** **0001**

ACCI, Age-Adjusted Charlson Comorbidity Index; CI, confidential interval; HGS, handgrip strength.

The receiver operating characteristic curve (ROC) was used to assess the area under the curve (AUC), sensitivity, and specificity of handgrip strength and ACCI in the prediction of overall survival and recurrence-free survival. A *p* value <0.05 was considered statistically significant and highlighted in bold text.

Moreover, the combined handgrip strength–ACCI model yielded the highest sensitivity and specificity, at 81.23% and 83.27% for overall survival and 80.54% and 82.62% for recurrence-free survival, respectively. By comparison, handgrip strength alone showed sensitivities and specificities of 65.61% and 68.24% for overall survival and 61.78% and 64.89% for recurrence-free survival (Figure 2 and Table 6). ACCI alone showed sensitivities and specificities of 72.34% and 74.36% for overall survival and 74.27% and 73.45% for recurrence-free survival (Figure 2 and Table 6). Time-dependent AUC analyses were further performed to compare the discriminative performance of handgrip strength, ACCI, and the combined handgrip strength–ACCI model at 1, 2, 3, 4, and 5 years. For overall survival, the combined model consistently achieved higher AUCs than either handgrip strength or ACCI alone (all *p* < 0.05) (Supplementary Table S1). Similarly, for recurrence-free survival, the combined model demonstrated significantly higher AUCs than either handgrip strength or ACCI alone (all *p* < 0.05) (Supplementary Table S2). These findings provide preliminary evidence that the combined model offers consistently improved discrimination compared with either individual marker.

## Discussion

In this prospective cohort of 783 patients with primary liver cancer undergoing curative-intent hepatectomy, we demonstrated that both reduced preoperative handgrip strength and a higher ACCI were independently associated with worse overall survival and recurrence-free survival. These findings supporting the concept that long-term survival outcomes after hepatectomy reflect not only tumor burden but also host reserve and comorbidity profiles. These findings extend prior evidence that comorbidity burden and functional impairment meaningfully shape prognosis after liver resection in primary liver cancer and suggest that a simple dual-domain assessment may be clinically valuable in routine surgical oncology practice.

A key observation is that integrating functional reserve (handgrip strength) with comorbidity burden (ACCI) yielded incremental prognostic discrimination beyond either marker alone. In Kaplan–Meier analyses, patients with concomitantly low handgrip strength and high ACCI experienced significantly poorer overall survival and recurrence-free survival than those with high handgrip strength and low ACCI. In ROC analyses, the combined model demonstrated significantly higher discrimination than either handgrip strength or ACCI alone for both overall survival and recurrence-free survival across the 1–5-year follow-up period. These findings strengthen the evidence that handgrip strength and ACCI provide complementary prognostic information and support the incremental discriminative value of their combined assessment. This pattern is consistent with prior studies showing that handgrip strength, especially when combined with systemic inflammation/nutritional indices, improves risk stratification in patients with liver cancer (12). Taken together, our results support a pragmatic framework in which handgrip strength provides a rapid “bedside” proxy of physiologic reserve, while ACCI captures accumulated chronic disease burden and age-related vulnerability, the two complementary dimensions that plausibly interact to influence postoperative recovery, recurrence tolerance, and mortality risk (16, 17).

Mechanistically, low handgrip strength likely reflects sarcopenia and impaired physical function, which are closely linked to systemic inflammation and cancer-related catabolism that accelerate muscle protein breakdown and reduce anabolic responsiveness (18–20). Concomitant malnutrition further worsens this phenotype by limiting substrate availability for muscle maintenance and repair, and inflammation–malnutrition synergy has been associated with substantially higher mortality risk in cancer populations (21–23). In our cohort, low handgrip strength was more common in older patients, females, and those with hypoalbuminemia and reduced total protein, suggesting convergence between functional decline and nutritional compromise in patients selected for hepatectomy. From a surgical stress perspective, reduced strength likely represents diminished physiologic reserve, predisposing patients to higher infection susceptibility, and impaired recovery trajectories after major abdominal surgery.

In parallel, a higher ACCI represents multimorbidity and age-related physiologic constraints that can increase perioperative complications, restrict eligibility for adjuvant or salvage treatments, and elevate competing non-cancer mortality, thereby worsening overall survival and potentially shortening the window for effective recurrence management (24–26). Notably, prior work in hepatocellular carcinoma demonstrated that ACCI predicts both short-term and long-term outcomes after hepatic resection, supporting standardized comorbidity scoring as a practical adjunct to tumor staging in surgical decision-making (7, 27). Taken together, the co-occurrence of low handgrip strength and high ACCI plausibly identifies a “double-hit” host-risk state—reduced functional reserve plus high comorbidity load—that intensifies vulnerability to surgical stress and limits therapeutic options after recurrence.

Recent consensus frameworks, including the Global Leadership Initiative on Malnutrition (GLIM), emphasize that malnutrition in cancer patients is a complex syndrome characterized by impaired nutritional status, reduced muscle function, inflammation, and disease burden. Previous studies have demonstrated that malnutrition and reduced muscle mass are associated with inferior outcomes after liver cancer treatment. However, nutritional assessment alone may not fully capture the vulnerability of patients undergoing major surgery. In this context, handgrip strength provides a simple assessment of functional reserve, whereas ACCI reflects age-related comorbidity burden. Their combination may provide a more comprehensive evaluation of host vulnerability by integrating nutritional-functional status with overall health condition (28, 29).

Several limitations should be acknowledged. First, the cutoff values of handgrip strength and ACCI were derived from the present cohort and were not tested against established literature-based thresholds. Future studies should validate these cutoffs using independent multicenter cohort studies. Second, the combined model showed higher discrimination than either marker alone; however, because external validation was not performed, these findings should be interpreted as preliminary evidence of incremental prognostic value. Third, although histological type was adjusted for in multivariable analyses, the cohort included predominantly HCC patients with a relatively small proportion of ICC patients. Therefore, we could not reliably perform adequately powered histology-specific subgroup analyses. Future multicenter studies with larger and more balanced HCC and ICC cohorts are required to determine whether the prognostic effects of handgrip strength and ACCI differ according to tumor subtypes. Finally, although time-dependent AUC analyses indicated that the combined handgrip strength–ACCI model demonstrated better discrimination than either handgrip strength or ACCI alone, these findings were derived from a single-center cohort, and independent multicenter external validation is required before clinical application.

## Conclusion

Preoperative handgrip strength and ACCI were independent predictors of overall survival and recurrence-free survival in patients with primary liver cancer undergoing hepatectomy, and the combined handgrip strength–ACCI model demonstrated improved discriminative performance compared with either marker alone. It may provide a practical approach to perioperative risk stratification, optimization of perioperative management, and individualized postoperative surveillance following liver resection.

## Data Availability

The original contributions presented in the study are included in the article/Supplementary Material, further inquiries can be directed to the corresponding authors.
