# Supplementary material for: Exploratory evaluation of combined handgrip strength and Age-Adjusted Charlson Comorbidity Index for prognosis in patients with primary liver cancer undergoing hepatectomy: a single-centre pilot study
Source: Front Med (Lausanne). 2026 Sep 8;13:1875463. doi: 10.3389/fmed.2026.1875463 (PMC13593476; doi:10.3389/fmed.2026.1875463)
Supplement: Supplementary file 1 [file Datasheet1.docx]

**Sulplementary materials**

**Table S1：The time-dependent AUCs for HGS, ACCI, and combined HGS–ACCI in the discrimination of overall survival of patients with primary liver cancer.**

| **Time** | **HGS (AUC, 95% CI)** | **ACCI (AUC, 95% CI)** | **HGS+ACCI (AUC, 95% CI)** | **P1-value** | **P2-value** |
| --- | --- | --- | --- | --- | --- |
| 1-year | 0.75 (0.61-0.89) | 0.74 (0.62-0.88) | 0.82 (0.65-0.93) | 0.001 | 0.002 |
| 2-year | 0.72 (0.63-0.83) | 0.76 (0.63-0.87) | 0.84 (0.63-0.92) | 0.005 | 0.004 |
| 3-year | 0.73 (0.59-0.84) | 0.84 (0.67-0.92) | 0.89 (0.68-0.95) | 0.012 | 0.023 |
| 4-year | 0.71 (0.56-0.82) | 0.75 (0.64-0.85) | 0.86 (0.71-0.93) | 0.002 | 0.001 |
| 5-year | 0.76 (0.62-0.88) | 0.82 (0.66-0.91) | 0.87 (0.72-0.96) | 0.007 | 0.026 |

**Notes:** Time-dependent AUCs with DeLong tests were used to estimate the discriminative ability of HGS, ACCI, and the combined HGS–ACCI model at 1, 2, 3, 4, and 5 years. P1-value: Differences in AUCs between the combined HGS–ACCI model and HGS; P2-value: Differences in AUCs between the combined HGS–ACCI model and ACCI. A p value <0.05 was considered statistically significant.

**Abbreviations:** ACCI: age-adjusted Charlson Comorbidity Index; AUC: area under the curve; CI: confidential interval; HGS: handgrip strength.

**Table S2：The time-dependent AUCs for HGS, ACCI, and combined HGS–ACCI in the discrimination of recurrence-free survival of patients with primary liver cancer.**

| **Time** | **HGS (AUC, 95% CI)** | **ACCI (AUC, 95% CI)** | **HGS+ACCI (AUC, 95% CI)** | **P1-value** | **P2-value** |
| --- | --- | --- | --- | --- | --- |
| 1-year | 0.64 (0.51-0.82) | 0.72 (0.61-0.89) | 0.82 (0.72-0.92) | 0.002 | 0.003 |
| 2-year | 0.62 (0.52-0.78) | 0.76 (0.64-0.91) | 0.78 (0.61-0.93) | 0.001 | 0.013 |
| 3-year | 0.63 (0.54-0.81) | 0.73 (0.62-0.90) | 0.80 (0.63-0.94) | 0.004 | 0.002 |
| 4-year | 0.65 (0.51-0.83) | 0.75 (0.61-0.92) | 0.83 (0.71-0.93) | 0.015 | 0.008 |
| 5-year | 0.68 (0.55-0.82) | 0.78 (0.67-0.93) | 0.81 (0.68-0.92) | 0.005 | 0.001 |

**Notes:** Time-dependent AUCs with DeLong tests were used to estimate the discriminative ability of HGS, ACCI, and the combined HGS–ACCI model at 1, 2, 3, 4, and 5 years. P1-value: Differences in AUCs between the combined HGS–ACCI model and HGS; P2-value: Differences in AUCs between the combined HGS–ACCI model and ACCI. A p value <0.05 was considered statistically significant.

**Abbreviations:** ACCI: age-adjusted Charlson Comorbidity Index; AUC: area under the curve; CI: confidential interval; HGS: handgrip strength.
